# Removal of deep-sea sponges by bottom trawling in the Flemish Cap area: conservation, ecology and economic assessment

**DOI:** 10.1038/s41598-019-52250-1

**Published:** 2019-11-01

**Authors:** C. K. Pham, F. J. Murillo, C. Lirette, M. Maldonado, A. Colaço, D. Ottaviani, E. Kenchington

**Affiliations:** 1 IMAR/OKEANOS - Universidade dos Açores, Departamento de Oceanografia e Pescas, 9901-862 Horta, Portugal; 2 Department of Fisheries and Oceans Canada, Bedford Institute of Oceanography, 1 Challenger Drive, Dartmouth, NS B2Y 4A2 Canada; 3 Department of Marine Ecology, Centre for Advanced Studies of Blanes (CEAB - CSIC), Acesso Cala St. Francesc 14, 17300 Blanes, Girona Spain; 4 Food and Agriculture Organization (FAO), Via delle Terme di Caracalla, 00153 Rome, Italy

**Keywords:** Conservation biology, Marine biology, Environmental impact

## Abstract

Deep-sea sponge grounds are vulnerable marine ecosystems, which through their benthic-pelagic coupling of nutrients, are of functional relevance to the deep-sea realm. The impact of fishing bycatch is here evaluated for the first time at a bathyal, sponge-dominated ecosystem in the high seas managed by the Northwest Atlantic Fisheries Organization. Sponge biomass surfaces created from research survey data using both random forest modeling and a gridded surface revealed 231,140 t of sponges in the area. About 65% of that biomass was protected by current fisheries closures. However, projections of trawling tracks estimated that the sponge biomass within them would be wiped out in just 1 year by the current level of fishing activity if directed on the sponges. Because these sponges filter 56,143 ± 15,047 million litres of seawater daily, consume 63.11 ± 11.83 t of organic carbon through respiration, and affect the turnover of several nitrogen nutrients, their removal would likely affect the delicate ecological equilibrium of the deep-sea benthic ecosystem. We estimated that, on Flemish Cap, the economic value associated with seawater filtration by the sponges is nearly double the market value of the fish catch. Hence, fishery closures are essential to reach sponge conservation goals as economic drivers cannot be relied upon.

## Introduction

Bottom trawling can have dramatic impacts on deep-sea habitats, where many benthic species are ill-adapted to physical disturbance, being highly susceptible to damage and removal as bycatch from bottom contact fishing gears. For example, a trawl passing over a pristine area of cold-water corals can catch up to 1.4 tons of coral per hour^1^, while more than 5 tons of sponge from a single research vessel trawl have been recorded to be lost from the sponge grounds on Flemish Cap in the northwest Atlantic^2^. The United Nations General Assembly (UNGA), through a series of resolutions to promote sustainable and responsible fisheries in marine ecosystems, has called upon States and Regional Fisheries Management Organizations and Arrangements (RFMO/As) to protect vulnerable marine ecosystems (VMEs) from destructive fishing practices. In particular, UNGA resolution 61/105 emphasizes the concept of significant adverse impacts (SAIs) of fishing on VMEs in relation to the need for management actions. To assist States and RFMO/As in implementing the UNGA resolutions, the Food and Agricultural Organization of the United Nations (FAO) produced international guidelines for the management of deep-sea fisheries in the high seas, and provided further direction on the identification of VMEs and what constitutes SAIs^3^. According to the FAO guidelines^3^ (hereafter referred to as ‘the guidelines’), SAIs are “*those that compromise ecosystem integrity (i.e. ecosystem structure or function) in a manner that; (i) impairs the ability of affected populations to replace themselves; (ii) degrades the long-term natural productivity of habitats; or (iii) causes, on more than a temporary basis, significant loss of species richness, habitat or community types*”^3^. The guidelines further elaborate that adverse impacts, in this context, produce negative effects on a VME resulting from damage caused during the operation of bottom-contact fishing gears. It is clear that ecosystem-level functions and processes are the relevant biotic scale, while SAI occur if ecosystem function is impaired and the long-term natural productivity degraded on more than a temporary basis. Ecosystem recovery following impacts is considered more than temporary if recovery takes more than 5 to 20 years^3^.

To date, RFMO/As have made progress in identifying VME indicator taxa and moving forward to protect VMEs from bottom-contact fisheries^4^. However, there has as of yet been no quantitative assessment of SAI of fishing on VMEs, despite the long history of research on trawling impacts on benthic ecosystems^5^. This is in a large part due to a lack of knowledge on the spatial distribution of both VMEs and fisheries in the high seas, and on the basic functional connections within and between deep-sea benthic communities.

**Figure 1:**
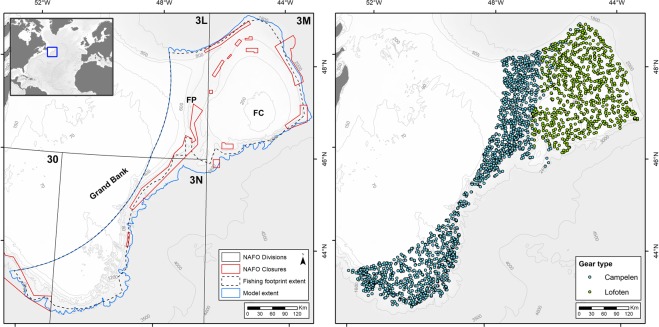
Location of the study area (left) that includes the Flemish Cap (FC), Flemish Pass (FP) and Grand Bank. NAFO statistical divisions (3 LMNO), NAFO fishing footprint area, the area of the distribution model and the NAFO areas closed to protect vulnerable marine ecosystems^50^ are indicated. Location of the groundfish surveys (2006–2010) conducted with two distinct trawl gear types that were used to estimate total sponge biomass (right). Maps were produced in ArcMap 10.2.2 software^46^.

Sponges are essential components of deep-sea ecosystems and are found in all oceans in a wide variety of settings on continental shelves and slopes, but also on seamounts, mounds, island slopes, and on the abyssal plain^6^. The three-dimensional structure created by sponge grounds provide habitats for a wide variety of organisms, many that are of commercial interest^7,8^. In fact, sponge grounds have been identified as biodiversity hotspots, comparable to tropical coral reefs, and are of significant ecological and economic value^6^. In addition to habitat formation, the filter-feeding capacity of sponges implies a large consumption of organic particulate carbon (grazing mostly on bacterioplankton and microplankton) and dissolved organic matter. Such a consumption has an impact not only on the benthic-pelagic coupling but also on the microbial loop itself^9–11^. Sponges may also have a significant impact on the benthic-pelagic coupling of inorganic nutrients, such as silicate, nitrate, nitrite, ammonium and phosphate^12^. Extensive, dense sponge grounds occur in deep-water habitats, but due to operational constraints most of our knowledge on the ecological functions of sponges have been derived from shallow-water species. The few investigations addressing the ecological functionality in relation to benthic-pelagic coupling of deep-water sponges have concentrated on a small number of species, including glass sponges *Aphrocallistes vastus* and *Rhabdocalyptus dawsoni*^9,13^, and demosponges *Geodia barretti, G. atlantica*, and *G. macandrewii*^10,11,14^. Consequently, the current understanding of the ecological role of deep-sea sponges is still quite incomplete. Extensive, dense aggregations of sponges — “*Geodia* grounds”—were recently described at bathyal depths in the Flemish Cap area, in the northwest Atlantic^2^. As a result, deep-sea sponge grounds have been recognized as VMEs by the Northwest Atlantic Fisheries Organization (NAFO). In 2012, NAFO closed a number of bathyal areas (Fig. 1) to protect vulnerable deep-water ecosystems from bottom fishing activities^15^, however portions of the *Geodia* grounds, and sponge communities on the shallower portions of Flemish Cap (Murillo *et al*. submitted), remain unprotected.

The objective of this study was to quantify, for the first time, the SAI of trawling on a deep-water ecosystem dominated by sponges. Through spatial modelling of the sponge biomass over the Flemish Cap area of NAFO, we were able to make a first quantitative assessment of sponge biomass removal by the international commercial trawling fleet from 2010–2012, using vessel monitoring system (VMS) data, and to estimate the degree of sponge protection provided by the current closed areas. At the same time, we were able to derive estimates of the impact of trawl sponge removals on benthic ecosystem functioning with respect to filtration, respiration, organic carbon assimilation and nitrogen cycling. We further undertook an economic evaluation of those impacts in terms of reduction of ecosystem function and we compared, in economic terms, the disruption of sponge water filtering activity due to trawling with the economic value of the commercial catch.

## Results

### Sponge biomass estimation

**Figure 2:**
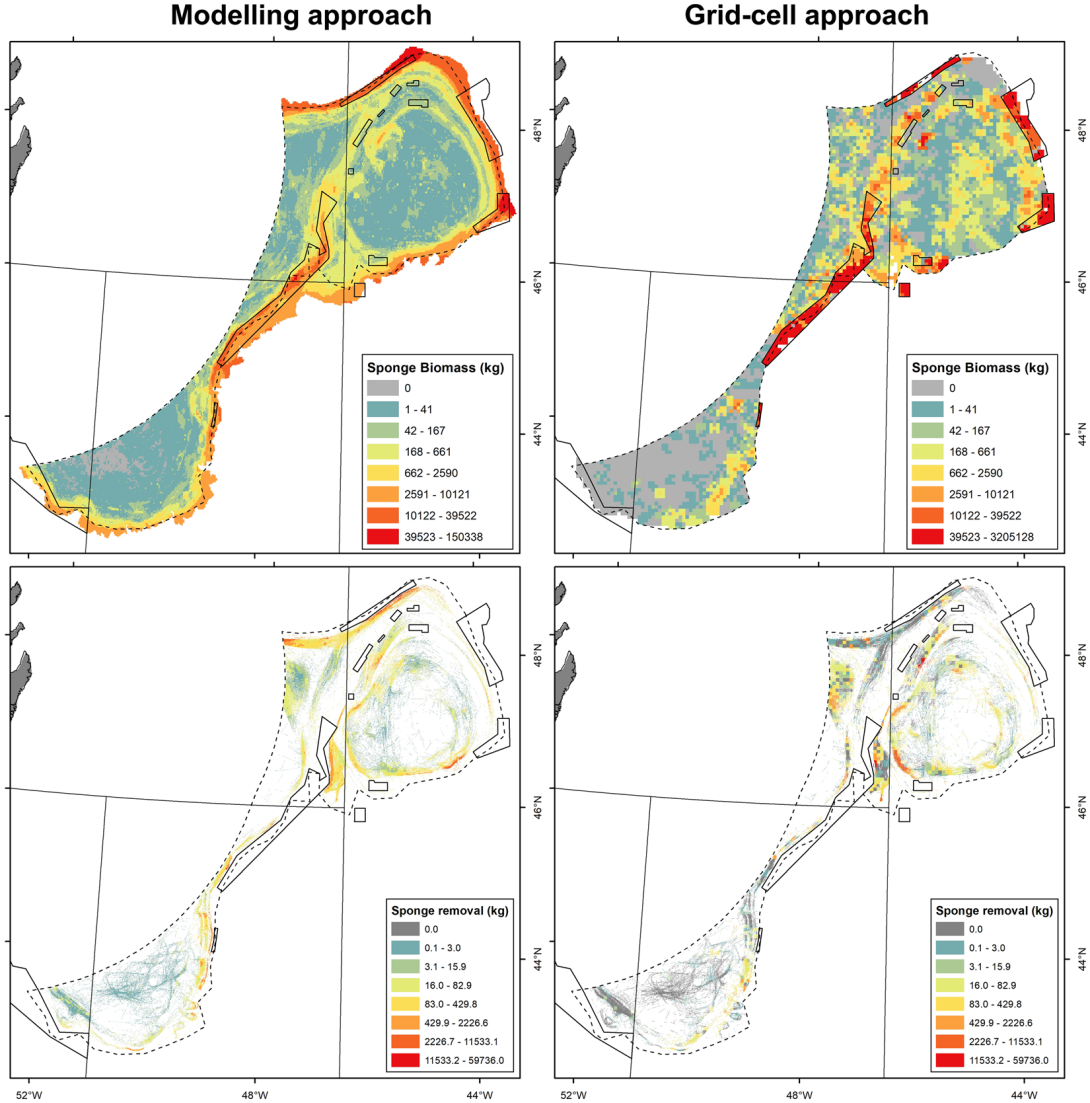
Total sponge biomass in wet weight based on biomass model (R^2^ = 0.40 ± 0.03) (top left) and the grid-cell (top right) approaches. The respective removal estimates by the fishing fleet operating in the Flemish Cap area between 2010 and 2012 are given in the bottom panels. Empty cells in the top right map indicate areas where no data were available to estimate sponge biomass for the grid cell. Zero values indicate areas where the mean biomass was zero (i.e., no catch of sponge). Dotted line indicates the NAFO fishing footprint. Maps were produced in ArcMap 10.2.2 software^46^.

Total wet weight biomass of sponges estimated from the modelling approach was 231,136 t, spread throughout a modelled area encompassing a total of 135,056.82 km^2^ of seabed and depths ranging between 50 and 2000 meters (Fig. 2; Table 1). Within the Flemish Cap ecosystem production unit (Division 3M), 147,837 t of sponge biomass was estimated. Overall, the closed areas protect 96,300 t of sponge biomass, 64% of which is protected within the Flemish Cap ecosystem production unit (Table 1).

**Table 1:** Surface area, sponge wet weight biomass and total removal in four NAFO Divisions estimated for the entire study area, the NAFO fishing footprint, and the areas closed to protect VMEs.

Spatial extent	Surface area (km^2^)			Biomass (t)		Fishing footprint (km^2^)	Removal (t)	
	Total area	Modelling approach	Grid-cell approach	Modelling approach	Grid-cell approach		Modelling approach	Grid-cell approach
**Modelled Area**	**135 056.82**	—	**121 959.17**	**231 136**	**121 967**	—	—	—
3L - Flemish Pass	26 802.12	—	25 869.34	28 060	24 814	—	—	—
3M - Flemish Cap	61 605.37	—	56 817.54	147 837	61 321	—	—	—
3N - Tail of Grand Bank	40 143.01	—	33 452.17	51 543	35 831	—	—	—
3O -	6 506.32	—	5 820.12	3 696	1	—	—	—
**Grid-cell Area**	**123 307.31**	**121 959.17**	—	**137 866**	**122 465**	—	—	—
3L - Flemish Pass	26 035.06	25 869.34	—	21 392	24 814	—	—	—
3M - Flemish Cap	57 312.36	56 817.54	—	85 429	61 819	—	—	—
3N - Tail of Grand Bank	33 899.54	33 452.17	—	28 739	35 831	—	—	—
3O -	6 060.35	5 820.12	—	2 306	1	—	—	—
**NAFO Fishing Footprint**	**120 047.80**	**119 246.07**	**118 613.24**	**116 143**	**81 169**	**22 765.18**	**4 815**	**661**
3L - Flemish Pass	25 292.40	25 125.42	25 243.03	16 573	12 431	6 518.49	1 095	207
3M - Flemish Cap	56 395.00	56 323.71	55 366.88	76 994	48 799	9 630.51	2 580	367
3N - Tail of Grand Bank	32 572.40	32 104.72	32 303.91	20 574	20 938	5 466.85	1 120	86
3O -	5 788.00	5 692.22	5 699.424	2 002	1	1 149.33	20	1
**Closures to Protect VMEs**	**26 513.84**	**12 830.09**	**12 304.11**	**96 300**	**118 562**	—	—	—
3L - Flemish Pass	2 736.02	2 736.02	2 726.62	11 930	24 062	—	—	—
3M - Flemish Cap	6 696.59	5 252.35	5 382.00	61 790	59 000	—	—	—
3N - Tail of Grand Bank	2 896.93	2 890.50	2 757.29	19 170	35 500	—	—	—
3O -	14 184.30	1 951.22	1 438.20	3 410	—	—	—	—

Using the grid-cell approach, total wet weight biomass of sponges was estimated to be 122,465 t for a smaller area of seabed (123,307.31 km^2^; Table 1). This is an expected result given that the fishing footprint only encompasses the shallower portions of some of the sponge grounds. Within a common NAFO fishing footprint area, the two approaches produced more similar estimates (Table 1). The modelling approach estimated a total sponge biomass of 116,143 t while the grid-cell approach estimated 81,169 t.

Within the NAFO fishing footprint area, both the modelling and the grid-cell approaches suggested that most of the sponge biomass (66% and 60%, respectively) was found within NAFO Division 3M (Flemish Cap). The remaining portion of the sponge biomass occurred mostly in Division 3L (Flemish Pass) and Division 3N (Tail of the Bank), while only a small portion (<2%) of the total biomass was found within Division 3O.

Throughout the modelled area, bottom zones closed to protect VMEs (n = 14) cover an area of 12,830.09 km^2^. However, when looking at the area that falls inside the NAFO fishing footprint, and so vulnerable to fishing threats if protections were not in place, the closed areas cover 7,884.2 km^2^ of seafloor, protecting sponge biomass of 56,800 to 77,466 t as estimated from the modelling and grid-cell methods, respectively. For both approaches, the majority of sponge biomass was located within four closed areas (Areas 2, 4, 5 and 6; Table 2).

**Table 2:** Sponge biomass estimated within each of the areas closed by NAFO^50^ to protect VMEs using both the modelling and grid-cell approaches for both the entire study area and the area limited to the NAFO fishing footprint.

Closed Area Code	Sponge biomass (t)			
	Total		NAFO fishing footprint	
	Model	Grid	Model	Grid
1	816	685	488	541
2	29 619	58 527	15 727	31 395
3	1 038	729	—	—
4	20 334	21 011	10 174	14 649
5	18 524	18 461	9 750	11 909
6	22 330	18 714	18 716	18 538
7	22	20	22	20
8	8	1	8	1
9	21	25	21	25
10	75	73	75	73
11	9	5	9	5
12	3	1	2	1
13	96	310	96	310
14	3 405	—	1 711	—
**Total**	**96 300**	**118 562**	**56 799**	**77 467**

### Commercial fishing trawl tracks

**Figure 3:**
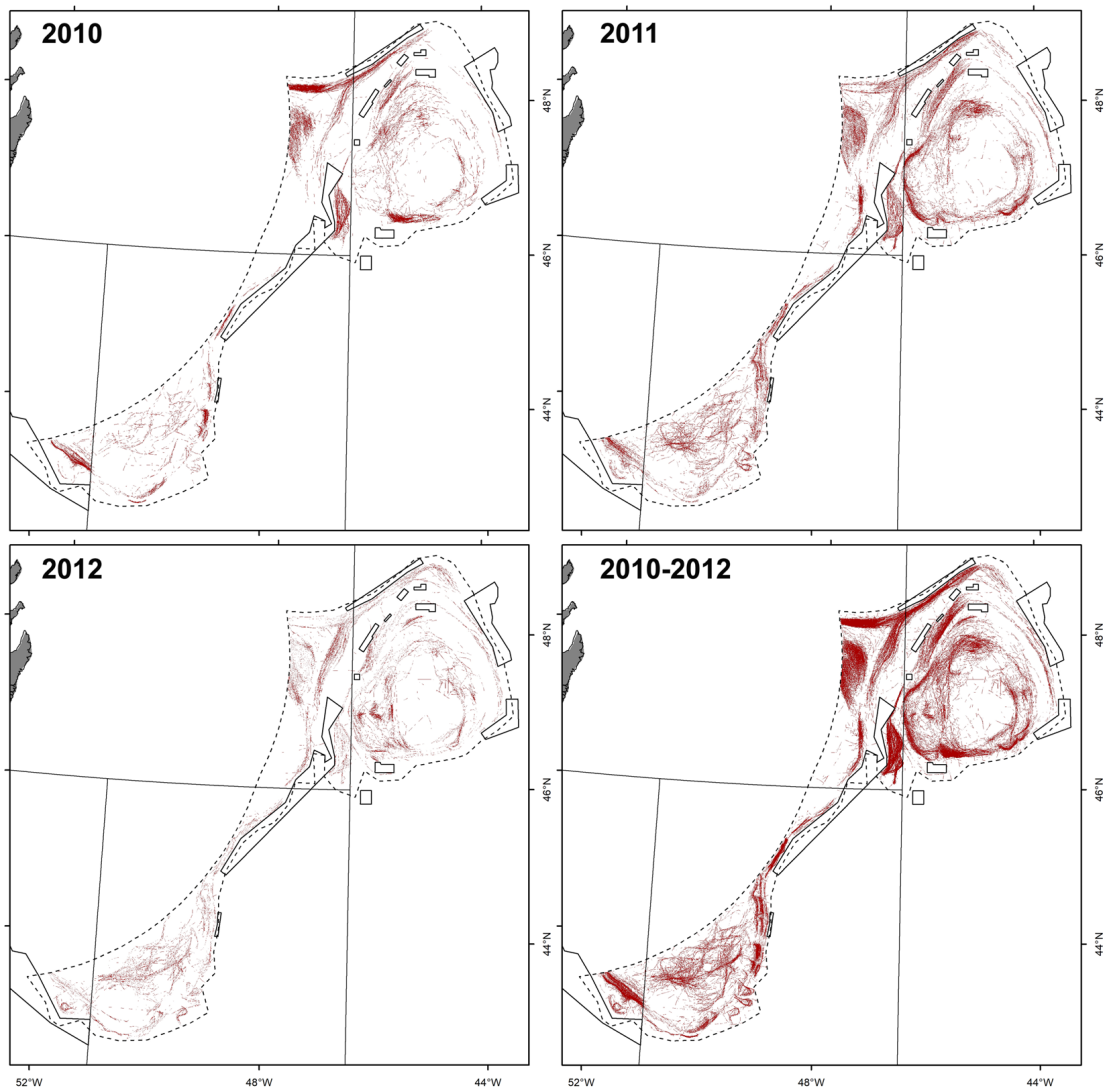
Commercial trawling locations (red) as determined by vessel monitoring system data in the NAFO fishing footprint^50^ (solid outline) between 2010 and 2012. Areas closed to protect VMEs are indicated^50^. Maps were produced in ArcMap 10.2.2 software^46^.

Between 2010 and 2012, a total of 12,942 separate trawl tracks were recorded within the study area, ranging from 3,113 to 6,293 trawl sets per year (Fig. 3). The total area of seabed impacted by this activity was estimated to cover 22,765.18 km^2^, thus covering 19% of the spatial extent of the total area (Table 1). This estimate represents the spatial extent of the trawling fleet on the seafloor and does not account for cumulative fishing effort occurring on a given area.

The majority of the trawling operations were found to occur within NAFO Division 3M (Flemish Cap) and Division 3L (Flemish Pass), accounting for 42% and 29% of the total fished area during this time frame, respectively. The fished area covered between 17% (3M and 3N) and 26% (3L) of the seafloor for each Division.

### Sponge biomass removal

The total removal of sponge biomass by the fishing fleet operating between 2010 and 2012 was estimated as 4,815 t using the modelling approach and 661 t using the grid-cell approach, with the former representing 4.4% of the total sponge wet weight biomass throughout the NAFO fishing footprint, and the latter representing 0.8% of the total sponge wet weight biomass estimated using the cell-grid approach. Although the fishing tracks were more prevalent in the Flemish Pass (Division 3L), both approaches suggested that the majority of sponge removal occurred on the Flemish Cap (Division 3M), where sponge biomass was the highest. Within Division 3M sponge biomass removals based on the modelling approach were estimated at 2,580 t, or 2.2% of total sponge biomass in the NAFO fishing footprint.

A simulation of 30 trawl tracks within the NAFO closures revealed that 884 t of sponges would be removed by these artificial trawls. The average (±SD) sponge removal per simulated trawling event was 29 ± 27 t. Therefore, it would take an estimated 2,947 directed trawling events to eliminate the entire sponge standing stock within the closures under assumption of non-overlapping fishing; a number corresponding to the average level of trawling effort observed between 2010 and 2012.

### Sponge ecosystem function

We estimated that the sponge grounds in the Flemish Cap area filter (±SD) 56,143 ± 15,047 million litres of seawater daily, with an associated respiration rate of 56.80 ± 10.65 t O_2_ day^−1^ (Table 3). This physiological activity involves a daily consumption of organic carbon of about 63.11 ± 11.83 t of C for the bottom water of an area encompassing 135,056.82 km^2^ of seafloor. In addition, this total sponge biomass is expected to have a daily uptake of ammonium (NH_4_^+^) of 0.02 t day^−1^ and about 0.41 t day^−1^ of nitrite (NO_2_^−^). The global release of nitrate (NO_3_^−^) by the sponge assemblage is also estimated at 6.5 t day^−1^.

**Table 3:** Sponge wet weight biomass estimated by the modelling approach and estimates of ecosystem functions in four NAFO Divisions.

Area	Biomass (t)	Filtration (x 10^6^ litre day^−1^)	Respiration (t O^2^ day^−1^)	Carbon consumption (t C day^−1^)	NH_4_^+^ (t day^−1^)	NO_2_^−^ (t day^−1^)	NO_3_^−^ release (t day^−1^)
3L - Flemish Pass	28 060	6 816 ± 1 827	6.90 ± 1.29	7.66 ± 1.44	0.002 ± 0.001	0.049 ± 0.016	0.79 ± 0.19
3M - Flemish Cap	147 837	35 910 ± 9 624	36.33 ± 6.81	40.37 ± 7.57	0.013 ± 0.003	0.259 ± 0.086	4.16 ± 1.00
3N - Tail of the Bank	51 543	12 520 ± 3 355	12.67 ± 2.37	14.07 ± 2.64	0.005 ± 0.001	0.090 ± 0.030	1.45 ± 0.35
3O -	3 696	898 ± 241	0.91 ± 0.17	1.01 ± 0.19	0.000 ± 0.000	0.006 ± 0.002	0.10 ± 0.03
**Total**	**231 136**	**56 143** ± **15 047**	**56.80** ± **10.65**	**63.11** ± **11.83**	**0.020** ± **0.005**	**0.406** ± **0.135**	**6.50** ± **1.57**

Sponge removal due to trawling activity resulted in a decrease in sponge biomass in the 3M Division of 2,580 t with a consequent estimated average decrease of the pumping capacity in the sponge ground of 627 ± 168 million litres day^−1^. High pumping rates and bacterial retention by the sponge indicate that, when in aggregations (i.e., sponge grounds), sponges are able to remove large quantities of bacteria and dissolved organic matter from the water column. This filtering capacity is similar to the infrastructure capacity of the Ashbridges Bay Treatment Plant (ABTP) operating in the city of Toronto serving a population of 1,524,000. The nominal treatment capacity of ABTP is of 818 million litres day^−1^, however the average daily flow rate recorded between 2015 and 2017 was of about 598 million litres day^−1^, thus substantially equivalent to the estimated filtering capacity by the removed sponge biomass in the 3M Division each day. The capital cost of a new UV disinfection facility, initiated in 2018, was reported as USD 182 million^16^. The forecasted operating cost of the Ashbridges Bay Treatment Plant, which included chlorination/dechlorination for primary effluent and UV disinfection for secondary effluent and bypass were determined in 2015 to be of about USD 1.7 million year^−1^ ^17^, therefore, operating costs over a 3-year period, which considered also the small inflation rate from 2015 to 2018 would be USD 5.4 million year^−1^. This is a very conservative cost estimate considering the importance of primary and secondary wastewater treatment and that the average operating costs, between 2015 and 2017, of the entire ABTP wastewater plant were reported to be of USD 45 million year^−1^ ^18^. Thus, comprehensively, the cost associated with a human engineered system capable of efficiently clearing bacteria from seawater at a rate similar to that of removed *Geodia* sponge biomass can be estimated at USD 187 million.

### Commercial catch and economic value in the NAFO area

Fish catch statistics in the examined NAFO subdivision 3M showed that the total catch from the trawl fleet cumulatively summed to 67,600 t between 2010 and 2012 with an average annual catch of about 22,500 t. The overall economic value for the three year period was estimated at USD 112 million (Table 4). However, considering the inflation rate from 2010 to 2018, the economic value of USD 112 million would be equivalent to a value of USD 125 million in 2018.

**Table 4:** Fish catch in quantity and value reported from trawling activities in the NAFO 3 M division.

Species common name	2010 (t)	2011 (t)	2012 (t)	Value 2010 (million USD)	Value 2011 (million USD)	Value 2012 (million USD)
Atlantic cod	9 564	15 960	14 500	18 735 451	33 102 275	24 822 180
Groundfish (NS)	4 658	3 786	3 838	5 181 757	4 291 103	4 894 955
Atlantic redfishes	4 233	5 133	338	5 436 154	8 669 485	534 995
Mixed species (marine fish nei)	3 783			2 033 120		
Greenland halibut	795		243	2 322 683		782 402
Shrimps (*Pandalus borealis*)	784			1 412 739		
TOTAL	23 817	24 879	18 919	35 121 904	46 062 863	31 034 532
Total adjusted for inflation rate	n.a.	n.a.	n.a.	40 445 346	51 421 495	33 942 451

It follows that the economic value of fish catch by trawling, based on ex-vessel fish and shellfish prices, is approximately half that of the economic value that can be associated to the loss of regulating function in the deep-sea ecosystem carried out by the estimated removal of sponge biomass by trawling between 2010–2012.

## Discussion

The present study provides the first assessment of the ecological and economic effects of sponge removal by commercial trawling activities operating in the Flemish Cap area. It also evaluates the efficacy of management actions put in place to protect the sponge-dominated communities in an Area Beyond National Jurisdiction. Although individual trawl sets operating in certain locations where shown to have high sponge catch, the overall magnitude of sponge removals in the NAFO Regulatory Area in the Flemish Cap region by the commercial fleet was limited. We found that the fishers are operating mainly in areas located outside of dense sponge grounds, as previously suggested by Murillo *et al*.^2^, and that the largest and densest sponge aggregations within the NAFO fishing footprint are currently protected by fisheries closures.

The results of this study highlight that the magnitude of impacts by bottom trawling on the deep-sea floor should be assessed at the scale of entire fishing fleets within ecosystems, because fishing pressure is not homogeneously distributed, making extrapolations based on small-scale studies highly uncertain. While both direct and indirect effects of trawling have been widely documented for deep-sea benthic communities^19^, quantifying impacts at larger spatial scales have been less common. Vessel monitoring system (VMS) data significantly advanced our understanding of actual fishing footprints, strengthening assessments of the impacts of trawling on seafloor habitats by providing a high level of spatial resolution^20^. In this study, VMS data from the bottom trawl fleet operating in the NAFO area of the Flemish Cap demonstrated that although the effort is considerable (~13,000 trawl sets between 2010 and 2012), it is concentrated in the same areas, impacting 19% of the entire extension of the NAFO fishing footprint based on historical records. Such detailed quantification of the fishing footprint coupled with extensive sponge biomass data obtained through systematic groundfish surveys (RV), allowed us to provide a reasonable estimate of sponge removal (hence direct mortality) by the bottom trawling fleet during those three years (between 661 and 4,815 t), representing 0.5–2.1% of total sponge biomass predicted to occur in the area (8–17% of sponges located outside closures and directly exposed to fishing pressure). The fisheries closures implemented by NAFO were confirmed to hold considerable sponge biomass that could in theory be completely removed by an estimated 2,947 non-overlapping trawling events. Given that the groundfish fleet operating in the Flemish Cap area from 2010 to 2012 performed between 3,113 to 6,293 trawl sets per year, it can be foreseen that the sponge fauna of the closed areas could potentially be eliminated in a single year of trawling activity. This illustrates that the sponge grounds in the closed areas could be quickly destroyed if protections were removed and fishing patterns changed.

It is important to highlight that the present assessment is based on sponge RV catch data collected between 2006–2010, in a general area already heavily trawled since the early 1960s^21^. Although it was beyond the scope of this study to determine sponge biomass of “pristine” grounds prior to trawling, a previous analysis of sediment cores did not observe the skeletal pieces (sterrasters) that characterize *Geodia* spp. in the current main fishing areas, suggesting that the spatial distribution of *Geodia* spp. sponge grounds on the Flemish Cap has never overlapped with the major fishing grounds^22^. This is different from the equivalent *Geodia* grounds (i.e., “ostur”) in the southern Barents Sea, where the trawl fishery shows a strong overlap with sponge grounds^23^.

The fact that the highest proportion of sponge biomass in this study region are found within the NAFO closures, does not mean that they are totally protected from fisheries effects. Re-suspended sediments can be carried into the closures given the high bottom currents in the region^24^, clogging and/or suffocating organisms that would otherwise have been out of the way of the trawl^25^. We do not have the necessary information to account for sedimentation-induced mortality in the present assessment of trawling mortality. However, considering the strong currents in some areas^24^, the sediments plumes are likely dispersing at a fast rate, impacting mostly the sponges found at the borders of closures adjacent to fishing areas. From the literature, it can be deduced that sediment deposition is, in general, considered quite a deleterious factor for sponges and that there is a growing concern about the increase of sediment deposition in the modern ocean due to a large variety of human-derived activities^25^.

For an in-depth understanding of SAI of bottom-contact fishing on sponges, knowledge of both gear selectivity and efficiency is necessary to draw conclusions from catch data (commercial or research vessel). The few studies that have examined both gear efficiency and selectivity of sponges, use experimental trawling to record removals and underwater video to record the true population size. For example, Moran & Stephenson^26^ quantified the catch of different epibenthic organisms greater than 20 cm, finding that less than 1% of “benthos” is retained by the gear. On the other hand, Sainsbury *et al*.^27^ found larger impacts on the northwest shelf of Australia, where a single pass of a fish trawl removed 90% of the large sponges. Therefore, it would appear that gear efficiencies may be anywhere from 1 to 90% for large sponges depending upon their shape and gear type. Due to the high uncertainty in sponge size structure coupled with high variability in gear efficiency and high incidental mortality for large sponges through detachment (of the size range of the *Geodia*-dominated grounds in the NAFO Fishing Footprint)^28^, we decided to use a 100% removal rate for a worst case assessment of total sponge removal in this region, in line with the precautionary approach to fisheries management. Our sponge biomass estimates also incorporated the catchability of the RV trawls into the resulting layers. True biomass could be significantly higher, although we assumed that the catchability is high (100%), based on the few *in situ* observations from the area. This is the same assumption of 100% catchability in the removal estimation from the commercial VMS tracks. Therefore, we have provided a conservative estimate of biomass and a worst-case scenario for removals which may serve to balance one another.

A complete assessment of SAI would also involve an evaluation of the time it takes for the ecosystem to recover^3^. Recovery of large sponges after trawling are addressed from few experimental studies, and little information exists on the growth and reproduction of most deep-sea sponges to adequately predict post-disturbance trajectories. The available studies indicate that deep-sea sponge grounds have comparatively low potential of recovering from physical disturbance events^29,30^, with depressed sponge densities even 13 years following disturbance^31^.

Marine sponges, and other filter-feeding animals in general, are able to pump enormous amounts of seawater, retaining with high efficiency dissolved organic matter, bacteria and microplankton^12^. The efficient performance of sponges as filter feeders has raised biotechnological interest about their potential to be assayed as biofilters able to remove pathogenic bacteria from seawater in particular systems^32^. When sponges occur aggregated at high densities, their retention efficiency translates into phenomenal amounts of organic C utilization^9,13,33^. Our modelled biomass for the entire area suggests that sponges filter about 56,143 ± 15,047 million litres of seawater daily and consume about 63.11 ± 11.83 t of organic carbon from the bottom water across an area encompassing 135,056.82 km^2^ of seafloor. The consumed carbon would derive from a mix of particulate (bacteria and microphytoplankton) and dissolved organic matter. A higher removal rate was reported by Kutti *et al*.^10^ for a marine protected area (MPA) off Norway, much smaller than our study area. The differences could be explained by our average sponge density (0.0017 kg m^−2^) throughout the region being three orders of magnitude lower (1.8 kg m^−2^) than in the MPA studied by Kutti *et al*.^10^. Although Geodiid sponges have been found in high densities on the Flemish Cap, occasionally peaking up to 10 individuals m^−2^ in Sackville Spur^34^, the patchy nature of their distribution makes the overall sponge density considerably lower over relatively large spatial scales, such as across the Sackville Spur closure (0.02 kg m^−2^) and our spatial 5 × 5 km cells, which hosted a maximum sponge density of 0.13 kg m^−2^. Although it is clear that the *Geodia* grounds on Flemish Cap appear to have a lower density in sponge biomass than equivalent *Geodia* grounds on the northern Norwegian continental shelf, we suspect that the low accuracy of the fluorescein method used by Kutti *et al*.^10^ when estimating pumping favored overestimation of the water flux and, consequently, magnified the derived C consumptions.

A review of the literature^35^ indicates that a sponge-dominated community can ingest 29–1970 mg C m^−2^ day^−1^, depending on the density of sponges and their metabolic rates, but also depending on the local availability and ratio of bacteria and DOM as carbon sources. The estimated daily consumption of C (63.11 ± 11.83 t) by the *Geodia* spp. population occurring over 135,056.82 km^2^ of seafloor renders an average ingestion of 0.46 mg C m^−2^ day^−1^, which is far below the minimum expected range. Yet, the phenomenal extension of the area where the sponges occur over the Flemish Cap makes the global C consumption from the bottom water a relevant regional issue. It is difficult to evaluate what the direct consequences of not removing 63 t of C daily in the form of bacteria and organic matter would be. Probably, the bottom water of that entire zone would be more anoxic, since the metabolism of bacteria is faster and more oxygen consuming than that of the sponges, which is among the lowest for filter-feeding invertebrates^36^. Sponges not only remove bacteria, but also compete for DOC with free-living bacteria, which, in the deep sea, are believed to be the main DOC consumers^37^. The tentative comparison between the filtering performance of sponges and equivalent human systems reveals the enormous energetic and economic cost that the removal of bacteria and DOC would involve if carried out by humans. In addition to C utilization, the sponge biomass has a daily uptake of ammonium (NH_4_^+^) of 0.02 t and 0.40 t of nitrite (NO_2_^−^), which are also crucial nutrients for bacterial proliferation. The sponges also contribute to a release of nitrate (NO_3_^−^) of about 6.5 t day^−1^, which enriches deep water with a nutrient critical to promote primary productivity when upwelled. If the fine equilibrium among sponges, bacteria and the flux of DOC and inorganic nutrient is perturbed, the consequences are difficult to predict but a shift in the deep-water system towards low oxygen or even anoxic scenarios appears quite plausible. It has already been hypothesized that the importance of sponges (*Geodia* spp.), through organic matter trapping and filtration, lies in the reduction of the detrital input to the adjacent soft-sediment communities, impacting the local biodiversity and carbon cycling^14^. It has also been hypothesized that the removal of the sponges will remove the structuring and functional effect of sponges, and that the increase in organic matter in the sediment if not immediately consumed by the infauna^38^, can create a local oxygen depletion. The oxygen depletion will diminish the potential of colonization due to avoidance by larvae^39^, and the habitat will take a long time to recover. If decreased oxygen availability at the bottom, the general complex food web associated with sponge grounds in the area^7,34^ may be affected.

The results of this study highlight the large extent and importance of sponge grounds in a productive fishing ground. Typically, data on benthic species has not been integrated directly into ecosystem production models directed at achieving an ecosystem approach to fisheries management. NAFO established a working group in 2008 whose long term objectives include developing a “Roadmap for Developing an Ecosystem Approach to Fisheries for NAFO”^40^. In their approach the VME closed areas were considered separately and were not integrated into the models which capture ecosystem production capacity^40^. An impediment to incorporating VMEs into such models has been the inability to equate data on the abundance and biomass of benthic taxa with ecosystem variables used in the models. Here we provide a suite of ecosystem functions produced by the sponge grounds within the Flemish Cap ecosystem production unit which may serve as an entry point for incorporating benthic habitats more directly into ecosystem approach frameworks. Operating in a broader but overlapping area, the Atlantic Ocean Research Alliance Working Group (AORA) on the Ecosystem Approach to Ocean Health and Stressors (EA2OHS WG), formed in response to the Galway Statement on Atlantic Ocean Cooperation, has created a framework characterizing all ocean-use sectors, stressors and ecosystem characteristics with the goal of relating them to one another and to the ecosystem goods and services they provide^41^. In that framework, quantifying ecosystem services is necessary for discussions of trade-offs among sectors, and our monetarization of disruption of regulating ecosystem services associated with the sponge grounds enables that discussion to take place. Our estimation of the monetary value of just a single function (filtration) showed that the cost associated with the loss of sponges is far more than the value of the fisheries (that *per se* will affect several ecosystem functions). Nevertheless, a simplistic economic valuation will not drive towards an appropriate level of protection of existing deep-sea sponge grounds and that fishing management tools, such as fishing closures, are essential to reach this conservation goal.

## Materials and Methods

### Study area

The study area lies in international waters off Newfoundland, Canada, within the 3LMNO management Divisions of NAFO (Fig. 1)^42^. Flemish Cap is considered both a bioregion and an ecosystem production unit, based on analyses of a suite of physiographic, oceanographic and biotic variables^43^, while it is treated as a discrete unit, NAFO Division 3M, for management of bottom fisheries. The Nose and Tail of the Grand Bank represent portions of a separate ecosystem production unit, the Grand Banks, which extends into the Canadian EEZ and includes the continental shelf in NAFO Divisions 3LNO^43^.

The benthic communities in this region have been well studied, and are typified by high biomass of large structure-forming demosponges, including *Geodia barretti*, *G. phlegraei*, *G. macandrewii* (Geodiidae), *Stryphnus fortis* and *Stelletta normani* (Ancorinidae). These sponges constitute more than 99% of the total invertebrate biomass over extensive areas^2,44^.

As a result of a moratorium that was enforced from 1999 to 2009 by NAFO due to a sharp decline in Atlantic cod (*Gadus morhua*) biomass in the early 1990s, commercial trawlers began targeting other species, such as Greenland halibut (*Reinhardtius hippoglossoides*), redfish (*Sebastes* spp.) and shrimp (*Pandalus borealis*). There is currently a moratorium on shrimp and pelagic redfish fisheries.

### Estimating total sponge biomass

Research vessel (RV) bottom trawl surveys are carried out annually for the assessment of fish stocks by Canada and EU/Spain/Portugal. Details on these multispecies surveys can be found elsewhere^2,44^. Sponge catch weight recorded at sea is estimated to the nearest 100 kg for the very large catches (some over 1 t) while smaller catches less than 200 kg are weighed at least to the nearest kilogram on both surveys.

After gathering data on sponge catch from 2769 survey catch records registered between 2006 and 2010 (Fig. 1) and assuming a 100% catchability two separate approaches where used to estimate sponge biomass: (1) biomass calculated from a distribution model using environmental predictors, the “modelling approach”; and (2) gridded biomass surface based on individual RV survey records, the “grid-cell approach”. The modelling approach is advantageous for obtaining a continuous biomass surface, allowing predictions in areas beyond the sampled locations based on environmental variables, and thereby capturing the full extent of the sponge grounds which in this area occur in deep waters where there are relatively few RV trawls. This allows our study to be placed into an ecosystem context. In contrast, the gridded biomass surface uses only the actual RV catch data to populate cells and so relies less on spatial interpolation/extrapolation. By using both approaches we are able to provide a range of potential impact of the fisheries on sponge ecosystem functioning, with the modelled biomass predictive surface likely a better representation of total sponge biomass in the region, while the gridded surface is likely more accurate at estimating removals by commercial trawling as it relies less on predictive inferences.

### Modelling sponge biomass using environmental predictors

In the “modelling approach” we used a random forest (RF) regression model^45^ to predict the distribution of the sponge biomass (wet weight) over the Flemish Cap and Nose and Tail of Grand Bank (Fig. 1). A total of 50 predictor variables, derived from different sources and with varying native spatial resolutions, were used to develop the model (Supplementary Table S1). These variables were chosen based on their availability, spatial coverage and statistical properties, and assumed relevance as proxies (based on previous studies) to the distribution of sponge biomass. Except depth and slope, the rest were spatially interpolated across the study area using ordinary kriging in ArcMap 10.2.2 software^46^. Spatial interpolation of these variables followed the approach described in Guijarro *et al*.^47^ for the Newfoundland and Labrador Region. All predictor layers were displayed in a raster format with geographic coordinates using the WGS 1984 datum and a ~0.013° cell size (approximately equal to 1 km horizontal resolution). The model was built in the statistical computing software program R version 3.3.1 using the ‘randomForest’ package^48^. Model parameters were evaluated using the ‘mlr’ package^49^. Models using 1000 regression trees, two randomly selected explanatory variables at each node, and the minimum size of terminal nodes provided the best results. Model performance was assessed in R using 10-fold cross validation repeated 10 times. The goodness-of-fit statistic R^2^ was used to measure the accuracy of the model.

The spatial distribution of data observations was mainly limited to above the 1500 m bathymetric contour. However, independent data, useful for validating the modelled results, showed that geodiid sponges do not appear to occur deeper than 2000 m^34^. Therefore, the spatial extent of the random forest model was restricted to the 2000 m depth contour. We refer to this predictive model spatial extent as the “modelled area”.

### Biomass estimation derived from research vessel catch data

In the “grid-cell approach”, data from the multispecies RV trawl surveys (Fig. 1) were used to build a gridded biomass surface (5 × 5 km). Details of the method used to create the surface are outlined in Cogswell *et al*.^50^, where for each grid cell the mean biomass was calculated based on all of the sponge RV catch data in each cell applied to the total cell area, and the biomass surface consisted of two merged grids (one clipped to and using data within closure areas and the other excluding data from within the closures). This approach allowed to capture the sharp transitions in biomass observed on the borders of some closed areas and therefore it provides a closer estimate of the actual sponge biomass along these areas (Supplementary Fig. S1). The value for cells with no RV trawl data is a function of a focal statistic calculation where the mean value of neighbouring populated grid cells (actual data) is used to populate the cell with no data.

Using this method and considering that a typical EU/Spanish trawl has a swept area of 39000 m^2^ (Lofoten gear) or 67000 m^2^ (Campelen gear)^44^, 373 RV Campelen trawl sets or 641 RV Lofoten trawl sets would be required to completely trawl a single grid cell without overlap. We applied the Campelen swept area to the NAFO areas 3LNO while the Lofoten swept area was used for the area 3 M, reflecting their usage in the different surveys (Fig. 1). The resultant gridded biomass layer contained cells with values representing the total sponge biomass (kg) assuming a 100% of catchability from both gears. The spatial extent of the “grid-cell approach” was restricted to areas surveyed by the RV surveys and is hereafter referred as the “grid-cell area”.

### Spatial distribution of fishing effort and total sponge removal

Commercial fishing trawl tracks (2010–2012) were derived from Vessel Monitoring System (VMS) data. Point data from these VMS records were created by the NAFO Secretariat by extracting “pings” with measured vessel speeds less than 6 knots, indicating that the vessels were likely fishing. Tracks were created where 2 or more consecutive “pings” at speeds <6 knots were observed for a single vessel and used to create separate lines for each of the trawls. The VMS data included groundfish and prawn fisheries for each of the years. In order to determine the footprint of each trawl event, a wingspread was attributed to simulate swept area. The wingspread of a typical commercial trawl is difficult to ascertain as this information is either not recorded, or not made public. We assumed a wingspread of 100 m, and that each trawl pass removed 100% of sponge biomass^27^ (see Discussion), similarly to the assumption used for estimating sponge biomass using RV catch data. The removal efficiency of a trawl is highly variable, therefore, we decided to assume that all sponges are removed (100%) in a single trawl pass, thus representing the worst-case scenario. This assumption is also based on the finding by Sainsbury *et al*.^25^ on the northwest shelf of Australia that a single pass of a fish trawl removed 90% of the large sponges. Therefore, the final fished area represents the total area of seafloor that was trawled at least once without accounting for subsequent trawling activities over the same area. Sponge biomass removal using the VMS tracks was estimated using both the “modelling” and “grid-cell approach”. To estimate total sponge removal for each grid cell, we first computed the percentage of area trawled in each cell and multiplied by the sponge biomass estimated per cell as described above. Total sponge biomass and removals were computed for the two different spatial extents: (1) the modelled area (135,056.82 km^2^) and (2) the grid-cell area which excludes some of the deeper extents of the sponge grounds (123,307.31 km^2^). A detailed explanation of the different spatial extents utilized in this study are presented in Supplementary Table S2.

### Predicting sponge removal in the absence of management measures

To further assess the effectiveness of fisheries closures for protecting sponge assemblages, we estimated the biomass removal of sponges by artificially simulating 30 trawl sets inside the closed areas (closed Areas 1 to 6, Table 2). The length of each trawl set mirrored a typical commercial fishing set (~18 nm covered, following an average vessel speed of 3 kt during 6 h) and were oriented following particular bathymetric contours similar to what the trawlers operating in this area would do. Due to the smaller size of closed Areas 1 and 3, the trawl sets simulated there covered a smaller distance (7 nm and 8 nm, respectively). Sponge removal was estimated following the same approaches described above.

### Sponge ecosystem function

About 90.2% of the sponge biomass in the sponge grounds of the study area are species from the genus *Geodia*, with dominance of the species *G. barretti*. Therefore, the functional role of the sponge assemblages in the Flemish Cap area can be approached reasonably through the functional parameters available for *Geodia barretti*. Estimates of total sponge biomass modelled for the entire study area (modelling approach) were converted into its functional values using rates of pumping, filtration, respiration, organic carbon consumption, and nitrogen (NH_4_^+^, NO_2_^−^, NO_3_^−^) flux per biomass unit. A variety of studies have described the pumping rate and basic physiology of *G. barretti*^10,11^, however Leys *et al*.^11^, uses the most accurate technique (Vectrino acoustic velocimeter). The parameters (mean ± SD) used herein for functional extrapolations are referred to sponge wet weight (WW): a filtration rate of 347 ± 93L sponge-kg^−1^ WW day^−1^, a respiration rate of 7.68 ± 1.44 μmol O_2_ sponge-g^−1^ WW day^−1^, an uptake of NH_4_^+^ of 0.02 ± 0.0026 μmol L^−1^ of filtered seawater and of NO_2_^−^ of 0.16 ± 0.05 μmol L^−1^, and a release of NO_3_^−^ of 1.87 ± 0.23 μmol L^−1^. As pumping rate given by Leys *et al*.^11^ were measured only for sponges that were previously demonstrated to be actively filtering by using fluorescein, the resulting values represented maximum pumping rates, with sponges filtering continuously over 24 h a day. Release of fluorescein around several *G. barretti* individuals at a *Geodia* spp. ground in the Barents Sea revealed that only some of the individuals were actively pumping water (H.T. Rapp., University of Bergen, personal communication). Therefore, we assumed that the daily pumping activity of the sponge individuals is about 70% of that considered in Leys *et al*.^11^. Again, to be cautious in our extrapolations, the respiration rate given by Leys *et al*.^11^ was further converted into carbon mineralization rate using a carbon to oxygen conversion ratio 0.9:1.0, which has been derived specifically for the sponge *Theonella swinhoei*^51^, rather than the conversion rate of 1:1 generally used for glucose respiration in animal cells. We applied these values to the total wet weight biomass for the area and total estimated wet weight biomass removed by the trawling in order to upscale total function provided by the sponges and the corresponding function loss caused through their removal by bottom trawling.

In order to assess the economic relevance associated with the loss of filtering capacity, we compared the pumping capacity of the lost sponge biomass due to trawling activities between 2010–2012 with the Ashbridges Bay Wastewater Treatment Plant (ABTP), the major sewage treatment facility of the city of Toronto, Ontario, Canada, characterized by similar filtering rates. The economic value associated to the loss of removed sponge biomass was estimated through a replacement cost valuation technique, which considered the operating costs of ABTP in effluent treatment and the capital cost of waste water plant infrastructure for the disinfection system of wastewater.

### Commercial catch and economic value in the NAFO area

The economic value of trawling activities in the Flemish Cap was quantified in order to provide a comparison with the loss of filtering capacity. We used the fish catch statistics reported by NAFO^52^ for the different divisions during the years 2010, 2011 and 2012. Due to the difficulty in differentiating domestic Canadian landings from international bottom trawl fleets, this quantification was only done for the NAFO Division 3M and restricted to certain species: Atlantic cod (*Gadus morhua*), Atlantic redfish (*Sebastes* spp.), Greenland halibut (*Reinhardtius hippoglossoides*), Shrimp (*Pandalus borealis*). A group of fish generically reported as groundfish and conservatively the unidentified group of mixed species (Marine fish not elsewhere included (nei)) were also included in the assessment.

The annual economic value of commercial catch in the NAFO 3M Division was assessed by multiplying the reported catch landings by the global reconstructed ex-vessel fish unit prices reported by Melnychuk *et al*.^53^ by species and species group for the analyzed years (2010, 2011 and 2012). Since the 3M Division is located in the high seas and commercial resources are harvested by different fishing fleets under different flag states, worldwide average fish and shellfish prices constitute a needed reference baseline. The annual economic value of commercial catches in the years 2010, 2011, and 2012 was referenced to 2018 by considering the average inflation rates recorded respectively over the periods 2010–2018 (1.78%), 2011–2018 (1.58%), 2012–2018 (1.50%) in order to obtain an updated market value of fish catch by bottom fisheries and compare it more directly with the estimated economic value of disruption of sponge ecological services in 2018.

### Ethics

The present work did not involve any contact or experimentation with the animals.

## Supplementary information

Supplementary Information

## Data Availability

The authors confirm that all data underlying the findings are fully available without restriction. Data from Canadian research vessel surveys are deposited in the Ocean Biogeographic Information System (OBIS) at http://obiscanada.marinebiodiversity.ca/or http://www.iobis.org/. Spanish/EU data are available at Figshare with the 10.6084/m9.figshare.1165479.
