# Author Correction: Removal of deep-sea sponges by bottom trawling in the Flemish Cap area: conservation, ecology and economic assessment

**DOI:** 10.1038/s41598-020-60908-4

**Published:** 2020-03-12

**Authors:** C. K. Pham, F. J. Murillo, C. Lirette, M. Maldonado, A. Colaço, D. Ottaviani, E. Kenchington

**Affiliations:** 1 IMAR/OKEANOS - Universidade dos Açores, Departamento de Oceanografia e Pescas, 9901-862 Horta, Portugal; 2 Department of Fisheries and Oceans Canada, Bedford Institute of Oceanography, 1 Challenger Drive, Dartmouth, NS B2Y 4A2 Canada; 3 Department of Marine Ecology, Centre for Advanced Studies of Blanes (CEAB - CSIC), Acesso Cala St. Francesc 14, 17300 Blanes, Girona Spain; 4 Food and Agriculture Organization (FAO), Via delle Terme di Caracalla, 00153 Rome, Italy

Correction to: *Scientific Reports* 10.1038/s41598-019-52250-1, published online 01 Novenber 2019

The original version of this Article contained errors in the Abstract.

“Sponge biomass surfaces created from research survey data using both random forest modeling and a gridded surface revealed 231,140 t of sponges in the area. About 65% of that biomass was protected by current fisheries closures.”

now reads:

“Sponge biomass surface created from research survey data using random forest modeling revealed 231,136 t of sponges in the area. About 42% of that biomass was protected by current fisheries closures.”

This error has now been corrected in the PDF and HTML versions of the Article.

