# Supplementary material for: Removal of deep-sea sponges by bottom trawling in the Flemish Cap area: conservation, ecology and economic assessment
Source: Sci Rep. 2019 Nov 1;9:15843. doi: 10.1038/s41598-019-52250-1 (PMC6825172; doi:10.1038/s41598-019-52250-1)
Supplement: Supplementary file 1 — Supplementary Information [file 41598_2019_52250_MOESM1_ESM.pdf]

SUPPLEMENTARY INFORMATION

Removal of deep-sea sponges by bottom trawling in the Flemish Cap area: conservation, ecology and economic assessment

Pham, C.K.[1*], Murillo, F.J.[2], Lirette, C.[2], Maldonado, M.[3], Colaço, A.[1], Ottaviani, D[4]., Kenchington, E.[2]

[1]IMAR/OKEANOS - Universidade dos Açores, Departamento de Oceanografia e Pescas, 9901-862 Horta, Portugal
[2]Department of Fisheries and Oceans Canada, Bedford Institute of Oceanography, 1 Challenger Drive, Dartmouth, NS, Canada, B2Y 4A2
[3]Department of Marine Ecology, Centre for Advanced Studies of Blanes (CEAB - CSIC), Acesso Cala St. Francesc 14, 17300 Blanes, Girona, Spain
[4]Food and Agriculture Organization (FAO), Via delle Terme di Caracalla, 00153 Rome, Italy
**Corresponding author: christopher.k.pham@uac.pt*

Table S1. Environmental variables and summary statistics used as predictor map layers in the random forest biomass distribution model. CHS_ABC: Canadian Hydrographic Service Atlantic Bathymetry Compilation; BNAM: Bedford Institute of Oceanography North Atlantic model (Wang et al., 2017); RSU-BIO: Remote Sensing Unit at the Bedford Institute of Oceanography; Max: Maximum; Min: Minimum.* N/A: Not applicable.

Variable	Metric	Unit	Native Resolution	Source
Depth	N/A	m	30 arc-sec	CHS_ABC
Slope	N/A	degrees	30 arc-sec	CHS_ABC
Bottom salinity	Max, mean, min, range	N/A	1/12th degree	BNAM
Bottom temperature	Max, mean, min, range	ºC	1/12th degree	BNAM
Bottom current speed	Max, mean, min, range	$m\ s^{-1}$	1/12th degree	BNAM
Bottom shear	Max, mean, min, range	Pa	1/12th degree	BNAM
Surface salinity	Max, mean, min, range	N/A	1/12th degree	BNAM
Surface temperature	Max, mean, min, range	ºC	1/12th degree	BNAM
Surface current speed	Max, mean, min, range	$m\ s^{-1}$	1/12th degree	BNAM
Fall mixed layer depth	Max	m	1/12th degree	BNAM
Winter mixed layer depth	Max	m	1/12th degree	BNAM
Spring mixed layer depth	Max	m	1/12th degree	BNAM
Summer mixed layer depth	Max	m	1/12th degree	BNAM
Fall primary production	Max, mean, min, range	$mg\ C\ m^{-2}\ day^{-1}$	9 km	RSU-BIO
Spring primary production	Max, mean, min, range	$mg\ C\ m^{-2}\ day^{-1}$	9 km	RSU-BIO
Summer primary production	Max, mean, min, range	$mg\ C\ m^{-2}\ day^{-1}$	9 km	RSU-BIO
Annual primary production	Max, mean, min, range	$mg\ C\ m^{-2}\ day^{-1}$	9 km	RSU-BIO

Wang, Z, Lu, Y, Greenan, B, Brickman, D, & DeTracey, B. An eddy-resolving North Atlantic model (BNAM) to support ocean monitoring. Can Tech Rep Hydrogr Ocean Sci 327: vii + 18p (2017).

Table S2. Details of the different spatial extents used to report sponge biomass and removal in this study.

**Name of Spatial Extent**	**Area (km^2)**	**Definition**	**Rationale**
Modelled Area	**135 056.82**	The NAFO area beyond national jurisdiction (ABNJ); NAFO Divisions 3LMNO; less than 2000 m	Biomass estimates from this area include the full spatial extent of the sponge grounds and hence more accurately reflects the sponge-dominated ecosystems in this area.
Grid-cell Area	**123 307.31**	The NAFO area beyond national jurisdiction (ABNJ); NAFO Divisions 3LMNO; delimited by the RV catch data	Biomass estimates from this area include the spatial extent of the RV survey data, including the NAFO fishing footprint and parts of the closures located outside the NAFO fishing footprint.
NAFO Fishing Footprint	**120 047.80**	The portion of the Modelled Area within the NAFO fishing footprint [50]	Biomass estimates from this area reflect the proportion of the biomass vulnerable to fishing impacts.
Areas Closed to Protect VMEs	**26 513.84**	The area within the areas closed by NAFO to protect VME[50], some of which lie all or in part outside of the NAFO Fishing Footprint.	Biomass estimates and derived functions from these areas reflect the protection at the ecosystem scale.

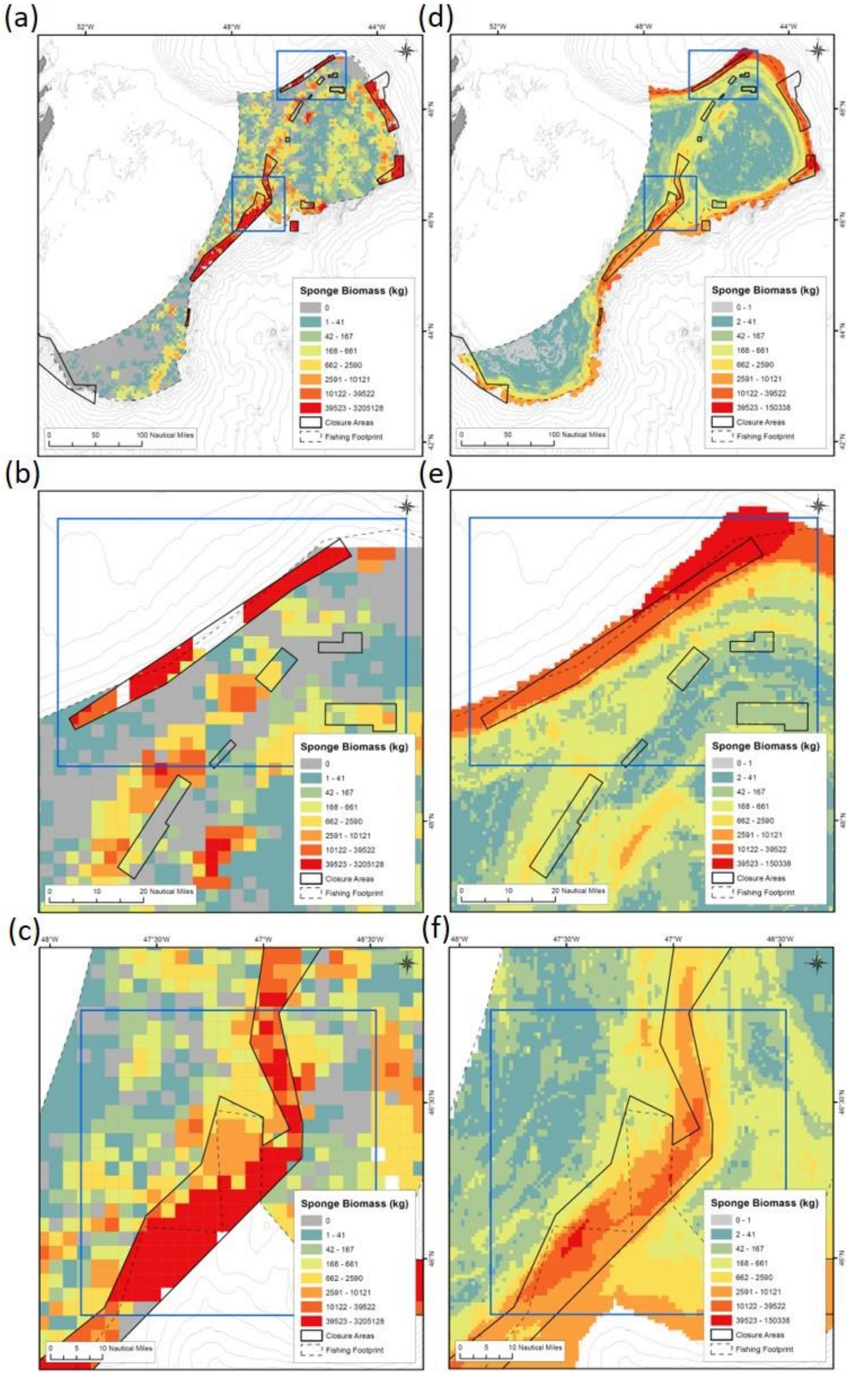

Fig. S1. (a) The sponge biomass surface from the “grid-cell approach” resulting from merging the “within closure areas biomass” and “outside closure areas biomass”. (b) and (c) are close ups of the Sackville Spur (upper blue box, in a) and Flemish Pass (lower blue box, in a) areas, showing the sharp delineation between high and low sponge biomass generated by merging the two biomass polygon layers. (d) The sponge biomass surface from the “modelling approach”. (e) and (f) are close ups of the Sackville Spur (upper blue box, in d) and Flemish Pass (lower blue box, in d), showing the gradual sponge biomass decrease between high sponge biomass inside the closures and low sponge biomass outside the closures inside the fishing footprint. Maps were produced in ArcMap 10.2.2 software[46]
